# Dramatic improvement in genome assembly achieved using doubled-haploid genomes

**DOI:** 10.1038/srep06780

**Published:** 2014-10-27

**Authors:** Hong Zhang, Engkong Tan, Yutaka Suzuki, Yusuke Hirose, Shigeharu Kinoshita, Hideyuki Okano, Jun Kudoh, Atsushi Shimizu, Kazuyoshi Saito, Shugo Watabe, Shuichi Asakawa

**Affiliations:** 1 Department of Aquatic Bioscience, Graduate School of Agricultural and Life Sciences, The University of Tokyo, Bunkyo, Tokyo 113-8657, Japan; 2 Department of Medical Genome Sciences, Graduate School of Frontier Sciences, The University of Tokyo, Kashiwa, Chiba 277-8562, Japan; 3 Department of Physiology, Keio University School of Medicine, Shinjuku, Tokyo 160-8582, Japan; 4 Laboratory of Gene Medicine, Keio University School of Medicine, Shinjuku, Tokyo 160-8582, Japan; 5 Division of Biomedical Information Analysis, Iwate Tohoku Medical Megabank Organization, Iwate Medical University, Shiwa-gun, Iwate 028-3694, Japan; 6 Akita Prefectural Institute of Fisheries, Oga, Akita 010-0531, Japan; 7 School of Marine Bioscience, Kitasato University, Sagamihara, Kanagawa 252-0373, Japan

## Abstract

Improvement in *de novo* assembly of large genomes is still to be desired. Here, we improved draft genome sequence quality by employing doubled-haploid individuals. We sequenced wildtype and doubled-haploid *Takifugu rubripes* genomes, under the same conditions, using the Illumina platform and assembled contigs with SOAPdenovo2. We observed 5.4-fold and 2.6-fold improvement in the sizes of the N50 contig and scaffold of doubled-haploid individuals, respectively, compared to the wildtype, indicating that the use of a doubled-haploid genome aids in accurate genome analysis.

The level of completion of genome sequencing for an organism is one of the most critical factors limiting progress in studies of its -omics, genetics, and evolution. Next-generation sequencing (NGS) technologies are expected to support whole-genome sequencing in nearly all species from mammals to microorganisms. However, the degree of assembly completion in higher organisms has often not been high; the draft genome sequences of higher organisms mostly consist of large numbers of contigs/scaffolds^1^,^2^,^3^,^4^,^5^,^6^,^7^,^8^,^9^, incorrect assemblies, and/or are missing some part of the genomes^10^.

Unlike the assembly of the longer read data obtained from Sanger or 454 pyrosequencing, the massive short reads from Illumina sequencers or their alternatives are usually processed by a de Bruijn graph–based algorithm. Therefore, many software applications have been developed, seeking to improve assembly accuracy with lower computer memory requirements^11^,^12^,^13^,^14^,^15^. However, little research has focused on efforts to improve biological starting material for genome assembly.

DNA polymorphisms in diploid species cause difficulty in assembling precise and long contiguous genome sequence data by shotgun sequencing using NGS technology or by the Sanger method. When similar, but not identical, genomic/cDNA sequences of an individual exist, it is often difficult to determine whether the difference is between polymorphisms or among repeated sequences in the genome. When a massively parallel short*-*read sequencer, such as Illumina, is employed and a de Bruijn graph is used for assembling, the single nucleotide polymorphisms (SNPs) or small indels cause branching of the graph, which complicates the assembly process^15^.

We aimed to improve genome assembly by enhancing the biological starting material, using fish as a model organism. *Takifugu rubripes* (torafugu) has the smallest genome size among vertebrates, which was initially sequenced in 2002^16^. In the latest assembly of the torafugu genome, 72% of scaffolds are located on the chromosome, but the remaining 28% have not yet been located or assigned^17^. The difficulty in assembling and scaffolding was partly due to the material for genome assembly, which was a natural heterozygous male individual. To avoid these problems, our previous report suggested that complete homozygous resources would improve the quality of genome assembly^18^.

In fish aquaculture, various reproductive technologies have been established to generate fish with favorable phenotypes, such as male/female^19^,^20^. Mitotic gynogenesis is one of the technologies^21^ by which we generated doubled-haploid (DH) torafugu individuals^18^.

Using the previously developed DH individuals^18^, we performed genome sequencing and compared the assembly results between wildtype (WT) torafugu and DH individuals. Here, by comparing the assemblies using the libraries from WT and DH torafugu individuals, we demonstrate improved quality of genome assembly using the DH torafugu individuals.

To allow for comparison of assemblies for WT and DH torafugu genomes, reads taken from a paired-end (PE) library (read length, 100 bp; mean insert size, 230 bp) of each torafugu individual (WT-1, WT-2, DH-1, and DH-2) were used for non-mixed assemblies. In a non-mixed assembly, reads from the library of a single individual were uploaded into a de Bruijn graph-based assembler. SOAPdenovo2^22^ was chosen to perform all assemblies in this study, because of its ability to produce large contigs with relatively few errors, as evaluated by Assemblathon 1 and GAGE^23^,^24^. The assembling was performed using four coverage levels (44×, 49×, 54×, and 59×) for each individual.

We evaluated the assemblies at each coverage level across four individuals with the metrics of N50 size and maximum length of contigs and scaffolds (Fig. 1a–d). With the increased inputs of different coverage levels, no significant differences in the sizes of contig N50 (Fig. 1a), contig maximum (Fig. 1b), or scaffold maximum (Fig. 1d) were observed for individuals, indicating that the scores, except for scaffold N50 (Fig. 1c), were saturated within the coverage range (44× to 59×). The two DH individuals (DH-1 and DH-2) showed the advantages of larger contigs and scaffolds. Compared with the contig N50 sizes in the two WT individuals (WT-1 and WT-2), the sizes in DH individuals extended from 4.7× to 6.0× (Fig. 1a). The scaffold N50 lengths increased from 2.2× to 3.2× for DH individuals (Fig. 1c). Additionally, the DHs showed increased maximum contig and scaffold lengths compared to the WT (Fig. 1b, d). The WT-1 individual produced larger contig N50 and scaffold N50 sizes (except for 59× in scaffold N50) than the WT-2 individual (Fig. 1a, c), which may be due to the presence of more homozygous regions in its genome.

The superiority of DH genomes can be interpreted by the de Bruijn graph algorithm, which is an algorithm feature of the assembler. The small polymorphisms such as SNPs cause branches and bubbles in de Bruijn graphs, resulting in ambiguity for contig formation. The assembler fails to disentangle the branches and bubbles and cannot determine the correct sequence connections; consequently, the assembler discontinues the sequence assembly. This obstruction explains why the polymorphic genomes of wild-type individuals yielded lower quality sequence results upon genome assembly.

We also performed another mode of assembly, mixed assembly, in which reads from other individuals were used for scaffolding with contigs formed in the non-mixed assemblies. Two trials were performed using mixed assembly. In the first trial, reads from two PE libraries (101-bp read length, 300-bp and 500-bp mean insert sizes) and two mate pair (MP) libraries (101-bp read length, 2-kb and 5-kb mean insert sizes) of DH-1 (Table 1) were used for scaffolding on the contigs formed from the 59× reads of each individual. In the second trial, reads from a PE library (101-bp read length, 400-bp mean insert size) and two MP libraries (76-bp read length, 2-kb and 5-kb mean insert sizes) of unrelated WT-3 were used (Table 1). We trimmed the PE reads to 100 bp and the MP reads to 75 bp, because we utilized data obtained from several different conditions before we performed the non-mixed assemblies. We used the same amounts of input libraries from two individuals (DH-1 and WT-3) in the scaffolding step, as shown in Table 2. Using MP reads of long insert size, the maximum scaffold sizes reached 2.6–7.2 Mb by scaffolding with the PE and MP data of DH-1, and scaffold sizes reached 3.8–7.5 Mb with those of WT-3. When we used the contig data of DH individuals, the scaffold N50 sizes were 1.9-fold to 2.7-fold larger than those obtained from the contig data of WT individuals. Consistent results were also observed for the longest scaffolds, where the improvement was 1.6-fold to 2.8-fold. These improvements were observed for the combinations of DH-1 contig and DH-1 MP/PE, DH-2 contig and DH-1 MP/PE, DH-1 contig and WT-3 MP/PE, and DH-2 contig and WT-3 MP/PE.

These results suggested that use of DH is very effective in generating longer contigs and scaffolds. Additionally, once better contig data are generated, the enhancement would extend beyond formation of scaffolds, even for MP/PE data obtained from different individuals. Interestingly, the sizes of scaffold N50 and scaffold maximum and the total residues of the combination of DH1 contig and WT-3 MP/PE were larger than those of DH1 contig and DH-1 MP/PE. This result might reflect the very reliable and robust results from the combination of DH1 contig and DH-1 MP/PE. In contrast, the result from DH-1 and WT-3 MP/PE might contain some incorrect scaffolding, because of the existence of two sets of haploid genomes in WT-3.

These comparisons evaluated the effects on assembling by mixing sequence data from different sources, in the event of insufficient DNA for library construction from doubled-haploid individuals. In the present study, we obtained sufficient DNA for the construction of various libraries from the 5-month-old gynogenetic individuals. However, the mito-gynogenetic larvae of some fish species often do not survive long or arrest development before hatching. Therefore, they do not synthesize a sufficient amount of DNA required for constructing Illumina MP libraries. Additionally, we could also utilize haploid (H) embryos, which were obtained by allowing the first egg cleavage during DH generation. Theoretically, the effects on the genome assembly caused by DH or H genomes are the same.

However, the amount of DNA from H embryos was also reduced. In order to compensate for the lack of DNA from DH and H individuals, mixed assembly, where the contig is generated from the limited amount of DNA from DH and H individuals, and scaffolding can be performed by using sufficient DNA from a WT individual. Thus, mixed assembly would be useful for genome sequencing of various fishes and other diploid organisms.

In results from the human genome project, LCRs (low copy repeats) and CNVs (copy number variations) were found to exist extensively in the human genome^25^,^26^, suggesting that LCRs and CNVs would also exist in the genomes of various organisms. CNVs are often associated with human genetic diseases^26^,^27^,^28^, suggesting that such regions are generally associated with various organism phenotypes.

The LCR regions were not trivial to read, even using BACs as the starting material for Sanger sequencing^25^,^29^. Furthermore, many CNVs are associated with LCRs^27^,^28^; therefore, it is more difficult to clarify the structure of such regions by sequencing diploid cells. For very polymorphic regions or CNV regions, the assembler may generate a pair of sequence data corresponding to each homologous chromosome. Such results may be desirable, but are often confusing, because it is difficult to determine whether such a pair of sequences exists as polymorphisms or repeats. When analyzing DH individuals, it is not necessary to consider polymorphisms, which dramatically simplifies the analysis of complicated genome regions.

The method described here is orthogonal to sequencing method developments and novel assembling algorithms. In the near future, scientists will be sequencing genomes of a wide variety of organisms^30^,^31^; consequently, to make such efforts more productive, our novel method should be principally considered.

## Methods

### Source of organisms

A female torafugu (WT-1) was purchased from a market in Akita Prefecture in 2010, and it was used for mito-gynogenesis. Female torafugus (WT-2 and WT-3) were purchased from markets in Akita Prefecture, Japan, in 2012 and Shimonoseki, Japan, in 2010, respectively. Those individuals were not related to the gynogenesis trial.

### Induction of mito-gynogenesis

To generate the DH torafugu larvae, mitotic gynogenesis, rather than meiotic gynogenesis, was induced by cold-shock treatments. The detailed processes of induction were described in our previous paper^18^. In brief, matured oocytes from WT-1 were fertilized by the sperm from a male torafugu, which was pretreated with UV-radiation dosages of 40, 80, and 160 mJ/cm^2^. Cold-shock treatments of 45 min duration were initiated 3 h post-fertilization at 0.6°C, 0.8°C, and 1.3°C for the three UV treatments. Eggs were incubated in aerated tanks with fresh seawater at 18.0°C before hatching.

### DNA sampling and whole-genome sequencing

Genomic DNA was sampled from five torafugu individuals according to the protocol for the DNeasy Blood & Tissue kit (Qiagen, Hilden, Germany). DNA libraries of these individuals were prepared according to the manufacturer's protocol (Illumina, San Diego, CA, USA) and sequenced by an NGS system (Illumina GA IIx and Illumina HiSeq 2000). Information regarding the raw sequence data from all sequencing trials for each individual is listed in Table 1. DH-1 and DH-2 individuals (mother, WT-1; 80 mJ/cm^2^ UV-treated sperm) hatched in June 2011 and were sampled 5 months later. The complete homozygosity of DH-1 was confirmed by both microsatellite genotyping and genome-wide SNP analyses, described in our previous paper^18^.

### Genome assemblies

Reads taken from a 230-bp PE library of WT-1, WT-2, DH-1, and DH-2 were used for contig and scaffold construction in non-mixed assemblies. We removed extra reads from the libraries of WT-1, WT-2, and DH-1 to ensure that all inputs from different individuals contained the same number of reads (236,267,482 reads). The SOAPdenovo2 assembler was chosen to perform *de novo* assembly, as it has been reported to surpass other assemblers on both assembled length and accuracy^22^. Considering the possible effect of coverage depth on assemblies, we set up four groups of reads for each individual to simulate a series of depths. The reads in the 44×, 49×, 54×, and 59× sets were taken from the 230-bp PE library. In each set, the number and the name of forward reads were identical to those of reverse reads. For each individual, four assemblies were generated with default settings under a k-mer value of 65.

In mixed scaffolding, reads from the libraries of DH-1 and WT-3 were used as inputs. To differentiate the difference in DH-1 and WT-3 read lengths, we programmed the assembler to use only the first 100 bp of each read from PE libraries and the first 75 bp of each read from MP libraries for scaffolding. Furthermore, we took half of the reads (42,428,578 reads) from the 300-bp PE library and another half from the 500-bp PE library of DH-1 to equal the number of reads in the 400-bp PE libraries (84,857,156 reads) of WT-3. After removal of duplicate reads in each MP library, the 2-kb and 5-kb MP libraries of both individuals were adjusted to contain the same number of reads (154,291,870 reads in 2-kb MP and 155,573,946 reads in 5-kb MP). With these pretreatments, the input reads from DH-1 and WT-3 for scaffolding were exactly the same in number (Table 2). Thus, the two trials were performed under the same conditions.

### Ethical note

All handling of fish was conducted in accordance with the “Guidelines for Proper Conduct of Animal Experiments” released by Science Council of Japan, and approved by Subcommittee on Institutional Animal Care and Use of Graduate School of Agricultural and Life Sciences, The University of Tokyo (permission # P14-952).

### Accession codes

The read data have been deposited in DDBJ under accession numbers DRR023068 to DRR023078.

## Figures and Tables

**Figure 1:**
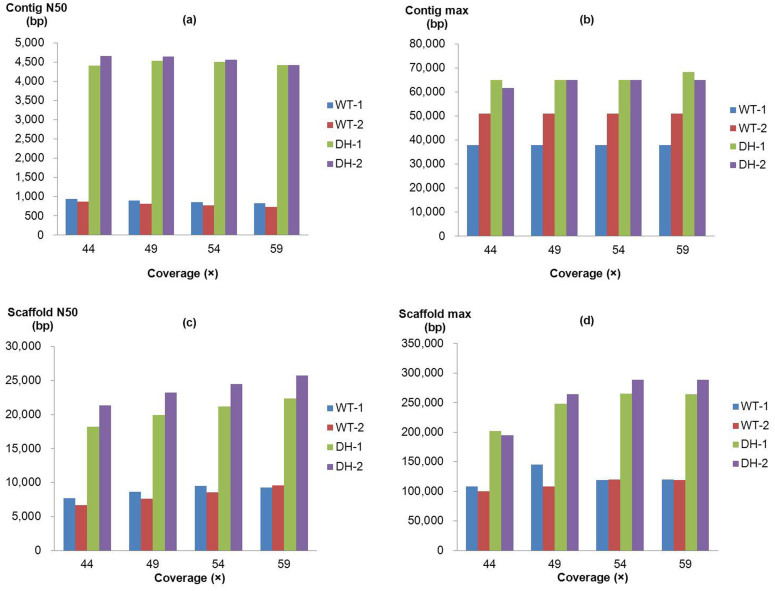
Comparison of assembly performance for wildtype and doubled-haploid genomes. The sizes of (a) contig N50, (b) contig max, (c) scaffold N50, and (d) scaffold max, including sizes of four individuals (WT-1, WT-2, DH-1, and DH-2) with different data coverage (44×, 49×, 54×, and 59×) are shown.

**Table 1:** DNA libraries and sequencing conditions

Individuals	Libraries	Instruments	Num. of seq	Length (bp)	Total residues (bp)
WT-1	230-bp PE	HiSeq 2000	251,423,596	100	25,142,359,600
WT-2	230-bp PE	HiSeq 2000	247,609,546	100	24,760,954,600
WT-3	400-bp PE	GAIIx	84,857,156	101	8,570,572,756
	2-kb MP	HiSeq 2000	278,642,344	76	21,176,818,144
	5-kb MP	HiSeq 2000	244,796,700	76	18,604,549,200
DH-1	300-bp PE	HiSeq 2000	283,351,680	101	28,618,519,680
	500-bp PE	HiSeq 2000	253,179,572	101	25,571,136,772
	230-bp PE	HiSeq 2000	245,201,650	100	24,520,165,000
	2-kb MP	HiSeq 2000	248,467,078	101	25,095,174,878
	5-kb MP	HiSeq 2000	339,507,094	101	34,290,216,494
DH-2	230-bp PE	HiSeq 2000	236,267,482	100	23,626,748,200

**Table 2:** Results of mixed scaffolding using paired reads of DH-1 and WT-3 libraries

Input libraries (Number of sequences for scaffolding)	Contigs	Scaffolds (bp)		
N50	Longest	Total residues		
DH-1300-bp PE^a^(42,428,578)500-bp PE^a^(42,428,578)2-kb MP^b^(154,291,870)5-kb MP^b^(155,573,946)	WT-1	353,902	2,553,311	450,109,058
WT-2	413,499	3,882,648	454,810,436	
DH-1	947,327	6,228,152	379,297,254	
DH-2	919,481	7,216,699	377,125,616	
WT-3400-bp PE^a^(84,857,156)2-kb MP^b^(154,291,870)5-kb MP^b^(155,573,946)	WT-1	449,077	3,817,845	448,973,730
WT-2	519,253	3,919,511	454,445,076	
DH-1	1,008,262	6,464,954	387,500,787	
DH-2	1,000,074	7,536,928	386,941,998	

aAll reads from the paired-end (PE) libraries were trimmed to 100 bp in length.

bAll reads from the mate pair (MP) libraries were trimmed to 75 bp in length.
